# Susceptibility of *Neisseria gonorrhoeae* to azithromycin and ceftriaxone in China: A retrospective study of national surveillance data from 2013 to 2016

**DOI:** 10.1371/journal.pmed.1002499

**Published:** 2018-02-06

**Authors:** Yue-Ping Yin, Yan Han, Xiu-Qin Dai, He-Ping Zheng, Shao-Chun Chen, Bang-Yong Zhu, Gang Yong, Na Zhong, Li-Hua Hu, Wen-Ling Cao, Zhong-Jie Zheng, Feng Wang, Qi Zhi, Xiao-Yu Zhu, Xiang-Sheng Chen

**Affiliations:** 1 National Center for STD Control, Chinese Center for Disease Control and Prevention, Nanjing, China; 2 Institute of Dermatology, Chinese Academy of Medical Sciences & Peking Union Medical College, Nanjing, China; 3 Dermatology Hospital, Southern Medical University, Guangzhou, China; 4 Guangdong Provincial Dermatology Hospital, Guangzhou, China; 5 Institute of Dermatology, Guangxi Autonomous Region, Nanning, China; 6 Sichuan Academy of Medical Sciences & Sichuan Provincial People’s Hospital, Chengdu, China; 7 Hainan Provincial Center for STD/Skin Disease Control and Prevention, Haikou, China; 8 Zhejiang Provincial Institute of Dermatology, Deqing, China; 9 Guangzhou Institute of Dermatology, Guangzhou, China; 10 Tianjin Center for Disease Control and Prevention, Tianjin, China; 11 Shenzhen Center for Chronic Disease Control, Shenzhen, China; 12 Xinjiang Center for Disease Control and Prevention, Urumqi, China; World Health Organization, SWITZERLAND

## Abstract

**Background:**

Gonorrhea remains one of the most common sexually transmitted diseases worldwide. Successful treatment has been hampered by emerging resistance to each of the antibiotics recommended as first-line therapies. We retrospectively analyzed the susceptibility of gonorrhea to azithromycin and ceftriaxone using data from the China Gonococcal Resistance Surveillance Programme (China-GRSP) in order to provide evidence for updating the treatment recommendations in China.

**Methods and findings:**

In this study, we included 3,849 isolates collected from patients with a confirmed positive *Neisseria gonorrhoeae* (*N*. *gonorrhoeae*) culture at clinic visits during the period of 1 January 2013 through 31 December 2016 in 7 provinces. Antimicrobial susceptibility testing of gonorrhea isolates using agar dilution was conducted to determine minimum inhibitory concentration (MIC). Resistance to azithromycin (RTA) was defined as MIC ≥ 1.0 mg/l, and decreased susceptibility to ceftriaxone (DSC) was defined as MIC ≥ 0.125 mg/l. The prevalence of isolates with RTA was 18.6% (710/3,827; 95% CI 17.4%–19.8%). The percentage of patients with DSC fluctuated between 9.7% and 12.2% over this period. The overall prevalence of isolates with both RTA and DSC was 2.3% (87/3,827; 95% CI 1.9%–2.8%) and it increased from 1.9% in 2013 to 3.3% in 2016 (chi-squared test for trend, *P =* 0.03). Study limitations include the retrospective study design and potential biases in the sample, which may overrepresent men with symptomatic infection, coastal residents, and people reporting as heterosexual.

**Conclusions:**

To our knowledge, this is the first national study on susceptibility of *N*. *gonorrhoeae* to azithromycin and ceftriaxone in China. Our findings indicate high rates of RTA and DSC from 2013 to 2016. Although dual therapy with azithromycin and ceftriaxone has been recommended by WHO and many countries to treat gonorrhea, reevaluation of this therapy is needed prior to its introduction in China.

## Abstract 2

In an observational study using data from the China Gonococcal Resistance Surveillance Programme, Yueping Yin estimate rates of reduced susceptibility to WHO-recommended antibiotics for Neisseria gonorrhoeae.

## Author summary

**Why was this study done?:**

- Antimicrobial resistance in *Neisseria gonorrhoeae* (*N*. *gonorrhoeae*) is a global threat in the control of this infection. China has one of the biggest antimicrobial consumption rates globally. However, China has limited data on antimicrobial resistance in gonorrhea.
- Systematic and timely collection and analysis of data on antimicrobial susceptibility of *N*. *gonorrhoeae* will inform the development of Chinese treatment guidelines and clinical practice standards.

**What did the researchers do and find?:**

- Minimum inhibitory concentrations (MICs) of azithromycin and ceftriaxone were determined for 3,849 clinical isolates from patients with gonorrhea who provided samples during the time period 2013 to 2016 in 7 provinces in China.
- Following the Clinical and Laboratory Standards Institute criteria, MIC values were used to define isolates as resistant to azithromycin and/or with decreased susceptibility to ceftriaxone.
- High prevalence of resistance to azithromycin (18.6%) and decreased susceptibility to ceftriaxone (10.8%) were found in our study population. The prevalence of concomitant resistance to azithromycin and decreased susceptibility to ceftriaxone increased from 1.9% in 2013 to 3.3% in 2016.

**What do these findings mean?:**

- *N*. *gonorrhoeae* strains resistant to azithromycin and/or with decreased susceptibility to ceftriaxone are prevalent in China, posing a challenge for gonorrhea control.
- The results can be used to inform national recommendations for *N*. *gonorrhoeae* treatment in China.

## Introduction

Gonorrhea, caused by *Neisseria gonorrhoeae* (*N*. *gonorrhoeae*), remains one of the most common sexually transmitted diseases (STDs) worldwide. In 2012, the World Health Organization (WHO) estimated 27 million prevalent and 78 million incident cases of gonorrhea among people 15–49 years old. Of these estimated numbers, 42.5% and 45%, respectively, occurred in the Western Pacific Region. China is the most populous country in this region and contributes substantially to the number of people with gonorrhea [1]. China reports approximately 115,000 new cases of gonorrhea annually. The incidence of gonorrhea has increased in recent years, and the infection has become one of the most common notifiable infectious diseases in the country [2]. Gonorrhea causes urethritis, cervicitis, pharyngitis, and anal infection. If left untreated, it can result in complications such as pelvic inflammatory disease in women [3] and infertility in both men and women [4]. Moreover, it increases the risk for HIV acquisition and transmission [5]. Control of gonorrhea in many countries largely relies on detection of cases followed by therapy to cure the infection and eliminate transmission to others. However, successful treatment has been hampered by emerging resistance to each of the antibiotics recommended as first-line therapies [6]. Latest in this trend are resistance and treatment failures for extended-spectrum cephalosporins, including ceftriaxone and cefixime [7,8]. Monotherapy with ceftriaxone (250 mg intramuscularly in a single dose) for uncomplicated gonococcal infections is still the recommended regimen in the current guidelines in China. To halt the development and spread of resistance, dual therapy consisting of ceftriaxone and azithromycin has been recommended as the first-line therapy in international and national guidelines [9–11]. However, the first treatment failure with dual therapy was reported in 2016 in the United Kingdom [12]. To provide evidence for updating China’s national treatment recommendations for gonorrhea, we analyzed data on the susceptibility to azithromycin and ceftriaxone of *N*. *gonorrhoeae* isolates collected in the China Gonococcal Resistance Surveillance Programme (China-GRSP).

## Methods

### Ethical clearance

The study was approved by the Medical Ethics Committee at the Institute of Dermatology, the Chinese Academy of Medical Sciences & Peking Union Medical College, and the National Center for Sexually Transmitted Disease Control (NCSTD) at Nanjing (approval number 2014-LS-026) for the use of anonymized samples and data collected during routine outpatient services to patients attending local dermatology and/or STD clinics.

### Study population

**Fig 1:**
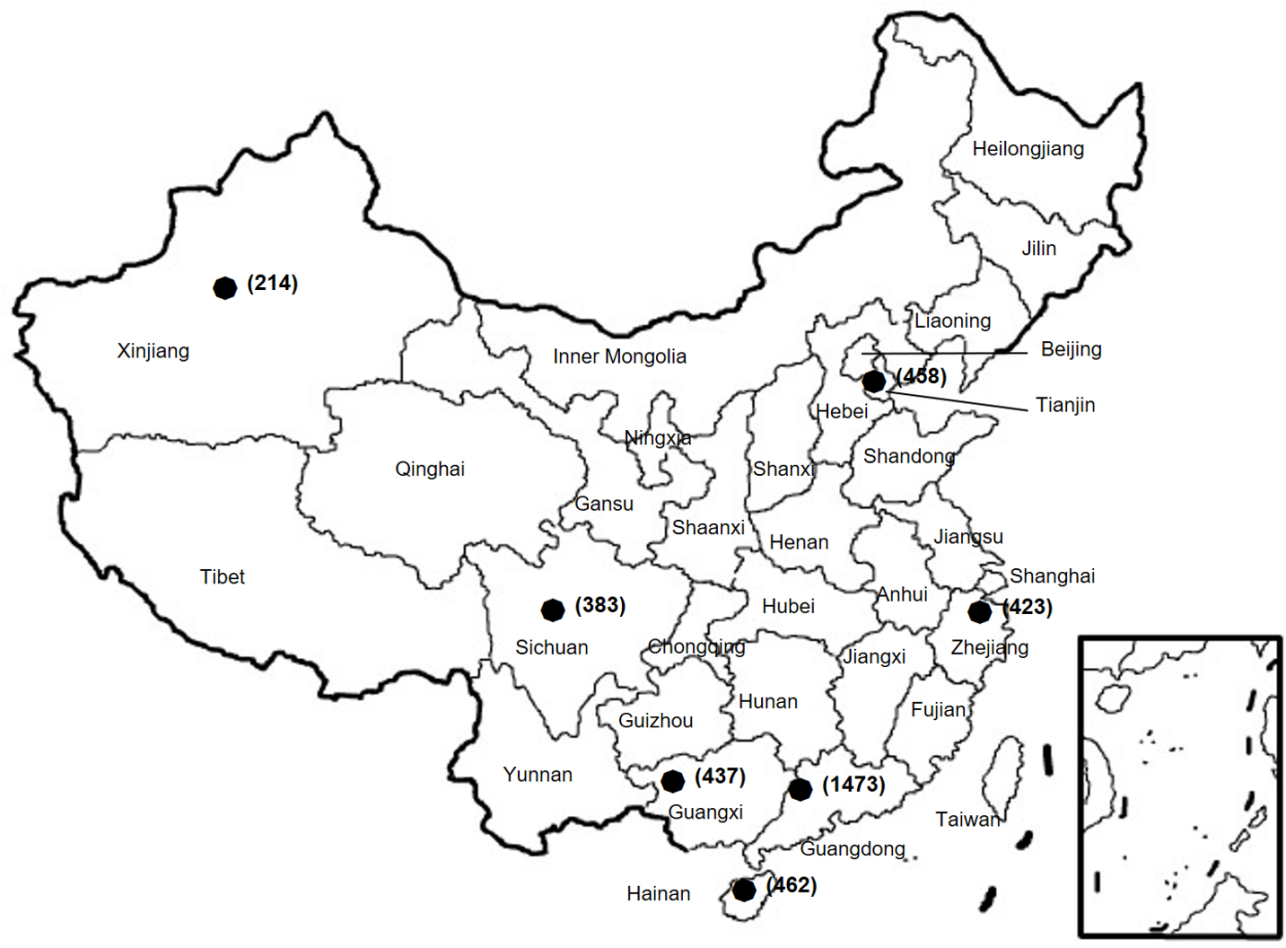
Geographic locations of the provinces where *N*. *gonorrhoeae* isolates were collected from patients. The number of isolates from each province is given in parentheses.

The current study was a retrospective study based on clinical isolates and data of patients with confirmed *N*. *gonorrhoea* in the China-GRSP. The China-GRSP was launched by the NCSTD as a collaboration project consisting of a few clinics in 1987. China joined the WHO Western Pacific Gonococcal Antimicrobial Susceptibility Programme (GASP) in 1992 and then expanded China-GRSP to more participating clinics in 9 provinces (Guangdong, Guangxi, Hainan, Zhejiang, Sichuan, Tianjin, Xinjiang, Shanghai, and Chongqing) in 2007. In 2013, China-GRSP started collecting data on azithromycin resistance. At each participating clinic, routine medical consultation was provided to patients. Cultures for *N*. *gonorrhoeae* were performed in patients with a positive risk assessment. Data from patients with confirmed *N*. *gonorrhoeae* from 1 January 2013 to 31 December 2016 were used in this study. The study focused on 7 provinces (Fig 1); Shanghai and Chongqing were not included in this study because these provinces did not collect data about azithromycin resistance. Selected demographic, behavioral, and clinical characteristics of the participants were extracted from outpatient medical records, i.e., from background information collected during the clinic visits.

### Isolation of *N*. *gonorrhoeae*

As a patient could be infected at different anatomical sites (cervix/vagina, pharynx, rectum, and urethra), samples from more than 1 site were collected, if indicated. Rectal and pharyngeal samples were generally not obtained from heterosexual males. Samples were inoculated into selective Thayer-Martin (TM) medium and cultured in an incubator at 36°C in 5%–10% CO_2_ for 24 to 48 hours. With the cultured isolate samples, probable *N*. *gonorrhoeae* was identified based on colonial morphology (growth of typical appearing colonies), Gram stain (Gram-negative), and oxidase reaction (oxidase-positive intracellular diplococci in stained smears). Sugar fermentation tests were further performed if the identification was ambiguous. The gonococcal isolates were further sub-cultured from the selective TM medium to a nonselective medium, and the confirmed colonies were suspended in skim milk and frozen to −70°C until shipped to provincial central STD laboratories for antimicrobial susceptibility testing.

### Antimicrobial susceptibility testing

Agar dilution antimicrobial susceptibility tests were conducted at the central STD laboratories of the participating provinces according to WHO recommendations [13]. Specifically, the isolates were cultured from frozen stocks onto selective TM medium and sub-cultured on GC medium base agar supplemented with hemoglobin powder and IsoVitaleX Enrichment (BD Diagnostics, Oxford, England) at 36°C with 5% to 10% CO_2_ for 18 to 20 hours at the central STD laboratories. The colonies were scraped, and suspensions of 10^7^ organisms per milliliter were prepared. Using a multipoint inoculator (10^4^ per point), the suspension was applied to antibiotic-containing medium (GC agar base supplemented with 10% defibrinated sheep blood; Nanjing Bianzhen Biotechnology, Nanjing, China) on a 9-cm diameter plate [14]. The plate was cultured at 36°C with 5% to 10% CO_2_ for 18 to 24 hours, and the growth of gonococci in different concentrations of antibiotic (0.03, 0.06, 0.125, 0.25, 0.5, 1.0, 2.0, 4.0, and 8.0 mg/l for azithromycin and 0.008, 0.015, 0.03, 0.06, 0.125, 0.25, 0.5, and 1.0 mg/l for ceftriaxone) was observed and recorded. The minimum inhibitory concentration (MIC) was defined as the lowest concentration of an antibiotic that inhibited the growth of gonococci. MIC interpretive criteria were in accordance with the Clinical and Laboratory Standards Institute to define resistance or decreased susceptibility [13,15]. For azithromycin, we categorized MIC values as susceptible (MIC ≤ 0.5 mg/l) or resistant (MIC ≥ 1.0 mg/l). For ceftriaxone, we categorized MICs as susceptible (MIC ≤ 0.06 mg/l) or decreased susceptibility (MIC ≥ 0.125 mg/l). For quality assurance, all the central STD laboratories participated in the external quality assurance program of the WHO Western Pacific GASP through the National STD Reference Laboratory in Nanjing.

### Statistical analyses

The development of the statistical analysis plan, including changes inspired by peer review, is described in S1 Text. Those patients whose isolates were successfully cultured and for which MICs for azithromycin and/or ceftriaxone were determined were included in the final analyses. In this study, we calculated the prevalence rates and their 95% confidence intervals (CIs) of *N*. *gonorrhoeae* resistance to azithromycin (RTA), decreased susceptibility to ceftriaxone (DSC), and both RTA and DSC (RTA/DSC). Age was described as median and 25%–75% interquartile range (IQR) because the age data followed a left-skewed distribution. MIC was described as geometric mean and 95% CI because MICs were multiple values determined by serial dilutions. For MICs determined as above or below a specific value, we used the midpoint value between this specific value and the value of the next higher or lower dilution for calculating the geometric means. Bivariate analyses were conducted by chi-squared test or chi-squared test for trend. Multinomial logistic regression analysis was conducted to explore the associations of variables with being infected with strains of *N*. *gonorrhoeae* with RTA, DSC, or RTA/DSC. Variables with a significance level of *P* < 0.10 in bivariate analyses were included in a multinomial logistic regression model. Adjusted odds ratios (AORs) and 95% CIs were estimated by adjusting for potential confounding factors that were included in the model. *P* ≤ 0.05 was set as the level of significance. Statistical analysis was done using SPSS for Windows (version 16.0; SPSS, Chicago, Illinois) and MedCalc for Windows (version 16.8; MedCalc Software, Mariakerke, Belgium).

The current study is reported as per Strengthening the Reporting of Observational Studies in Epidemiology (STROBE) guidelines (S1 Checklist).

## Results

### Baseline characteristics of patients

A total of 3,849 *N*. *gonorrhoeae* isolates were collected from 3,849 patients (males, 91.1%; females, 8.9%). Data on repeat infections in individuals were not available due to anonymization of the dataset during the study years. Of the 3,503 males who provided information on sexual orientation (99.9% of the male total), 3,451 (98.5%) were heterosexual and 52 (1.5%) were homosexual or bisexual. The median age was 31 years (IQR 26–40 years) for males and 31 years (IQR 25–43 years) for females. The majority (92.8%) of the participants were of Han ethnicity, and 50.2% were from the provinces along the coast, where more cases of gonorrhea were reported than in other areas [16]. Previous infection with gonorrhea was reported in 15.0% of patients. Among the heterosexual patients, the majority of the isolates were from the urethra (99.4%) in males and the cervix or urethra (96.2%) in females. No patients were found to have a positive *N*. *gonorrhoeae* isolate at more than 1 anatomical site.

### Azithromycin susceptibility

**Fig 2:**
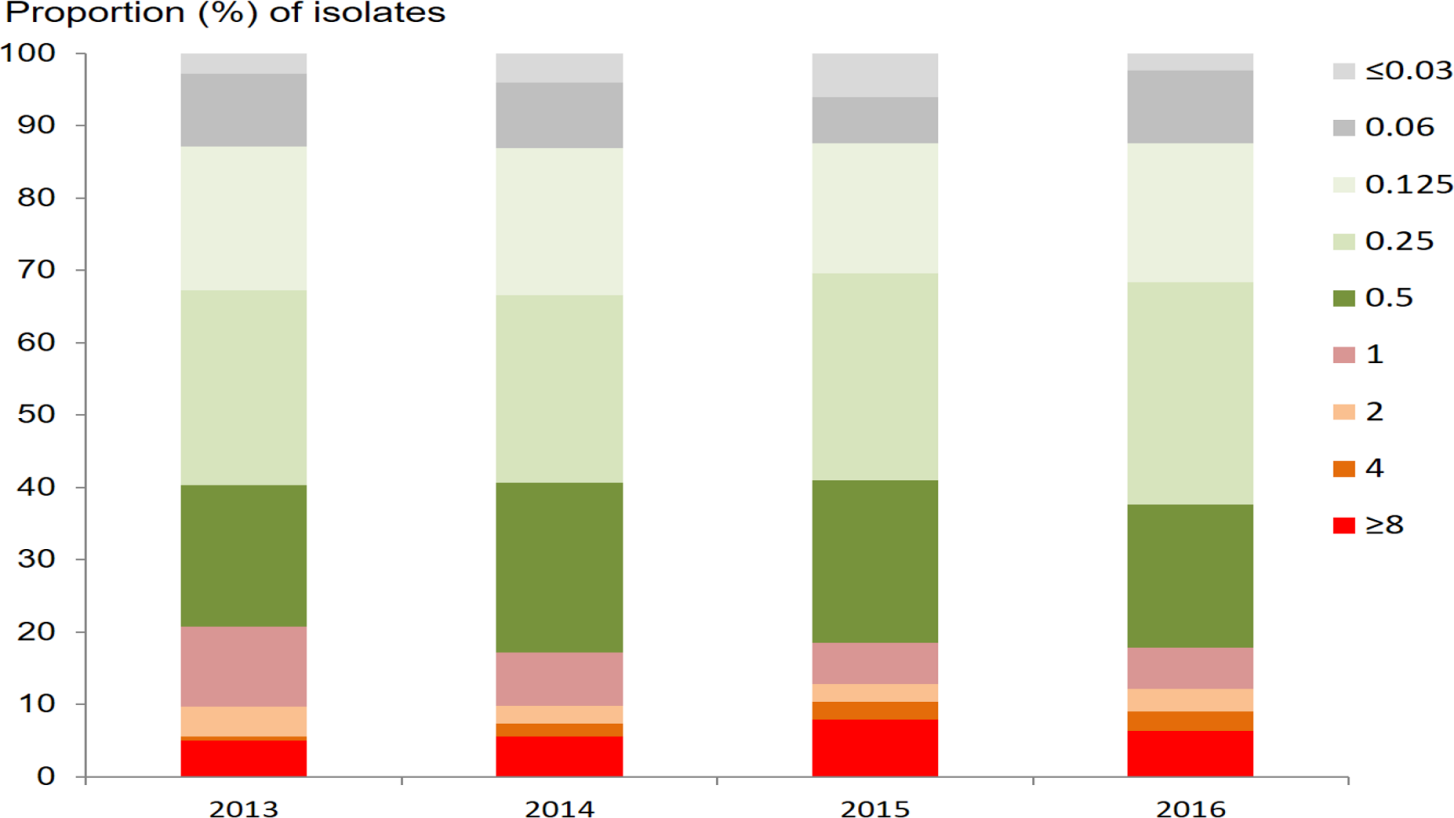
Proportion of *N*. *gonorrhoeae* isolates with different minimum inhibitory concentrations (mg/l) for azithromycin, by year.

Out of 3,849 *N*. *gonorrhoeae* isolates, 3,827 (99.4%) and 3,849 (100.0%) had MIC results for azithromycin and ceftriaxone, respectively. The geometric mean azithromycin MIC was 0.32 mg/l (95% CI 0.31–0.34 mg/l), with a range of ≤0.05 to ≥8 mg/l. The prevalence of RTA (defined as MIC ≥ 1.0 mg/l) was 18.6% (710/3,827; 95% CI 17.4%–19.8%). The proportions of the isolates with different MICs of azithromycin by year are shown in Fig 2. Neither the mean MIC nor the percentage of resistant strains appeared to change during the 2013–2016 study period, remaining between 0.31 mg/l and 0.34 mg/l and between 17.1% and 20.8%, respectively. Multinomial regression analysis (Table 1) indicates that RTA was significantly associated with younger age (AOR 0.99; 95% CI 0.98–1.00; *P* = 0.043) and being female (AOR 1.50; 95% CI 1.13–2.00; *P* = 0.005, compared with males).

**Table 1:** Associations between resistance to azithromycin or decreased susceptibility to ceftriaxone and demographic and clinical characteristics (multinomial regression analysis*).

Characteristics	Resistance to azithromycin (MIC ≥ 1.0 mg/l)**		Decreased susceptibility to ceftriaxone (MIC ≥ 0.125 mg/l)		Resistance to azithromycin and decreased susceptibility to ceftriaxone	
	Prevalence (*n/N*)	AOR (95% CI)	Prevalence (*n/N*)	AOR (95% CI)	Prevalence (*n/N*)	AOR (95% CI)
Age group (years)	――	0.99 (0.98–1.00)*	――	1.00 (0.98–1.01)	――	0.99 (0.97–1.01)
Sex						
Male	17.9 (624/3,486)	Reference	11.1 (389/3,507)	Reference	2.2 (77/3,486)	Reference
Female	25.2 (86/341)	1.50 (1.13–2.00)	8.2 (28/342)	0.77 (0.47–1.27)	2.9 (10/341)	1.42 (0.71–2.84)
Sexual orientation						
Heterosexual	18.6 (699/3,763)	Reference	10.9 (412/3,784)	Reference	2.3 (86/3,677)	Reference
Homosexual or bisexual	15.4 (8/52)	0.77 (0.34–1.74)	7.7 (4/52)	0.45 (0.14–1.48)	1.9 (1/52)	1.08 (0.14–8.12)
Previous gonococcal infection						
Yes	21.2 (122/576)	1.12 (0.87–1.43)	7.1 (41/577)	0.68 (0.45–1.03)	2.3 (13/576)	0.78 (0.41–1.46)
No	18.1 (587/3,243)	Reference	11.5 (374/3,264)	Reference	2.3 (73/3,243)	Reference
Residence along the coast						
Yes	17.1 (331/1,934)	1.00 (0.83–1.20)	12.9 (249/1,934)	1.84 (1.44–2.36)	1.7 (32/1,902)	0.56 (0.35–0.88)
No	20.0 (379/1,893)	Reference	8.8 (168/1,915)	Reference	2.9 (55/1,838)	Reference

*Reference value in the multinomial regression analysis: neither resistance to azithromycin nor decreased susceptibility to ceftriaxone.

**22 results for azithromycin MIC are missing.

AOR, adjusted odds ratio; CI, confidence interval; MIC, minimum inhibitory concentration.

### Ceftriaxone susceptibility

**Fig 3:**
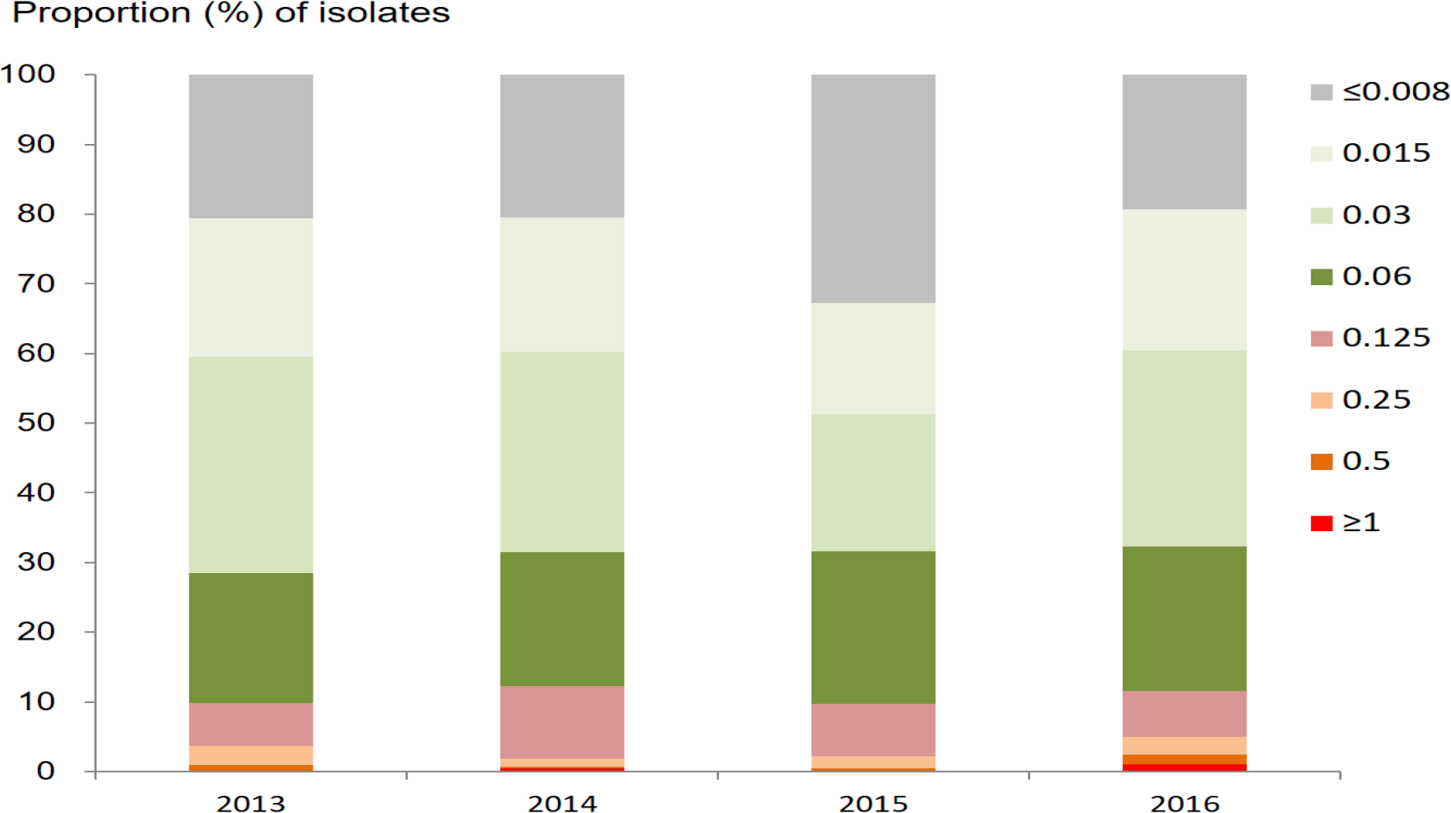
Proportion of *N*. *gonorrhoeae* isolates with different minimum inhibitory concentrations (mg/l) for ceftriaxone, by year.

The geometric mean ceftriaxone MIC was 0.025 mg/l (95% CI 0.024–0.026 mg/l), with a range of ≤0.008 to ≥1.0 mg/l. The prevalence of DSC (defined as MIC ≥ 0.125 mg/l) was 10.8% (417/3,849; 95% CI 9.9%–11.9%). The proportions of isolates with different MICs for ceftriaxone are shown by year in Fig 3. The percentage with DSC fluctuated between 9.7% and 12.2% over the period from 2013 to 2016. In the multinomial analysis (Table 1), DSC was significantly associated with residence in areas along the coast (AOR 1.84; 95% CI 1.44–2.36; *P* < 0.001, compared with residence in other areas).

### Resistance to azithromycin and decreased susceptibility to ceftriaxone

**Fig 4:**
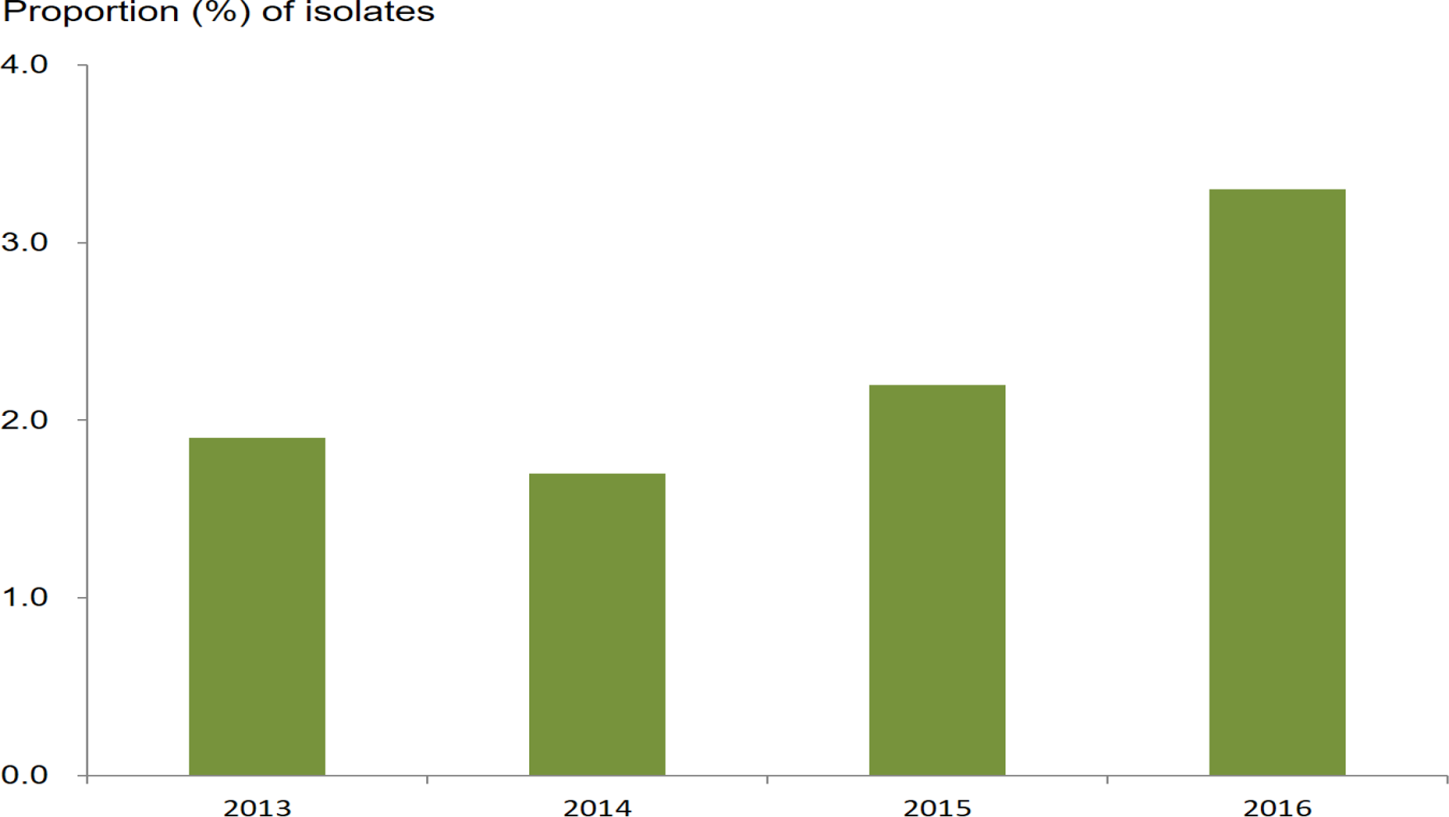
Proportion of *N*. *gonorrhoeae* isolates with resistance to azithromycin (MIC ≥ 1.0 mg/l) and decreased susceptibility to ceftriaxone (MIC ≥ 0.125 mg/l) from 2013 to 2016. MIC, minimum inhibitory concentration.

The proportion of isolates resistant to azithromycin in those with DSC (21.0%, 87/415; 95% CI 17.3%–25.1%) was not statistically different from the proportion of isolates resistant to azithromycin in those susceptible to ceftriaxone (18.3%, 623/3,412; 95% CI 17.0%–19.6%) (chi-squared = 1.79; *P* = 0.18; Table 2). The prevalence of isolates with RTA/DSC was 2.3% (87/3,827; 95% CI 1.9%–2.8%). The prevalence increased from 1.9% (18/928; 95% CI 1.2%–3.0%) in 2013 to 3.3% (32/981; 95% CI 2.3%–4.6%) in 2016 (chi-squared for trend = 4.78; *P* = 0.03) (Fig 4). Multinomial regression analysis (Table 1) indicates that residents in areas along the coast are less likely to be infected with strains with RTA/DSC (AOR 0.56; 95% CI 0.35–0.88; *P* = 0.013).

**Table 2:** Number and percentage of isolates susceptible or resistant to azithromycin by susceptibility breakpoints of ceftriaxone.

Ceftriaxone	Azithromycin*		
	Resistant (MIC ≥ 1.0 mg/l)	Susceptible (MIC ≤ 0.5 mg/l)	Total
Susceptible (MIC ≤ 0.06 mg/l)	623 (18.3%)	2,789 (81.7%)	3,412 (100.0%)
Decreased susceptibility (MIC ≥ 0.125 mg/l)	87 (21.0%)	328 (79.0%)	415 (100.0%)
Total	710 (18.6%)	3,117 (81.4%)	3,827 (100.0%)

*There were 22 isolates without MIC data for azithromycin.

MIC, minimum inhibitory concentration.

## Discussion

The current study represents, to our knowledge, the first national surveillance on trends of susceptibility of *N*. *gonorrhoeae* to azithromycin and ceftriaxone over time in China. In addition, associations between susceptibility data and demographic and clinical characteristics of study participants were examined. Our findings indicate that the rate of dual RTA and DSC increased between 2013 and 2016. The prevalences of *N*. *gonorrhoeae* RTA and DSC found in this study in China were higher than those reported in many countries, including in Europe and the Americas [17–27] (Table 3). A high prevalence of RTA or DSC has also been reported in Japan, although the sample size of the available study was relatively small (less than 700 isolates in total over 3 years) [27]. It is unclear whether circulating gonococcal strains with altered susceptibility to the key antibiotic agents in China acquired these mutations through domestic selective pressure or whether the strains have been imported. Antimicrobial resistant gonorrhea was documented first in Asia [28,29], then emerged in other areas in the world. Although azithromycin monotherapy is not recommended for gonorrhea treatment in China, this antibiotic is widely used for treatment of chlamydial infection according to the national STD treatment guidelines [30]. The prevalence of chlamydial infection is much higher than that of gonorrhea both among high-risk groups, such as female sex workers [31,32] and men who have sex with men [33], and in the general population [34] in China. Overuse of macrolides for chlamydia and other infections could facilitate emergence of resistance in *N*. *gonorrhoeae* [35]. Studies in China have reported that a high percentage (>50%) of outpatient visits result in prescription of antibiotics [36,37].

**Table 3:** Prevalence of resistance to azithromycin or decreased susceptibility to ceftriaxone among *N*. *gonorrhoeae* isolates collected from 2013 to 2016 in different countries.

Country/region by year	Resistance to azithromycin (MIC ≥ 1.0 mg/l)		Decreased susceptibility to ceftriaxone (MIC ≥ 0.125 mg/l)	
	Number of isolates	Percentage	Number of isolates	Percentage
United States				
2013 [17]	5,945	3.6	5,945	0.1
2014 [17]	5,093	7.1	5,093	0.1
Canada				
2014 [18]			3,809	0.05
European Union				
2013 [19]	1,994	5.4	1,932	0.4*
2014 [20]	2,147	7.9	2,015	0.25*
United Kingdom				
2013 [21]	1,750	1.6	1,750	0.2
2014 [21]	1,568	1.0	1,568	0.0
2015 [21]	1,699	9.8	1,699	0.0
Germany				
2010–2015 [22]	266	7.1		
Australia				
2013 [23]	4,897	2.1	4,897	0.6
2014 [24]	4,804	2.5	4,804	0.6
2015 [25]	5,411	2.6	5,411	0.1
Zimbabwe				
2015–2016 [26]			388	0.0
Japan				
2013 [27]	241	22.4**	241	31.5**
2014 [27]	192	14.1**	192	32.8**
2015 [27]	238	18.8**	238	29.4**
Our study in China				
2013–2016	3,827	18.6	3,849	10.8

*MIC > 0.125 mg/l for ceftriaxone was used in this study.

**These rate data came from personal communication with the corresponding author (Prof. Mitsuru Yasuda, Department of Urology, Gifu University Hospital) of the cited study.

MIC, minimum inhibitory concentration.

WHO and many countries have recommended dual antimicrobial therapy with ceftriaxone plus azithromycin for people with symptomatic and asymptomatic gonorrhea [9–11]. However, despite the high prevalence of DSC (more than 10%) in the current study, treatment failure of ceftriaxone has not been documented in China. It is important that clinicians be on high alert to recognize gonorrhea treatment failures so that they can be reported promptly to public health officials. The high and increasing prevalence of RTA/DSC found in the current study suggests the need for further consideration and validation of an appropriate regimen for treatment of gonorrhea in China.

Socioeconomic, behavioral, and clinical factors associated with antimicrobial resistance have been investigated by previous studies in China and other countries [38–40]. Interestingly, the factors independently associated with RTA and DSC were not the same in our study. Female patients had a higher risk of infection with an azithromycin-resistant strain than males. This finding was consistent with that reported in Amsterdam [41] but different from that reported in Shanghai, where male sex was a significant predictor of tetracycline resistance [38]. Our finding of an association of younger age with RTA is different from that reported in Shanghai, where probable resistance to ceftriaxone was most common among those in older age groups [38]. In addition, in our study, DSC was more likely to occur in areas along the coast, but RTA/DSC was more prevalent in other areas. These associations may be related to transmission dynamics linked to the sexual networks of different populations or in different areas. In addition, the resistance patterns are also dependent on the population seeking care in the sentinel clinics. Further studies on genomic epidemiology and sexual networks among people infected with resistant strains are needed. In contrast to the expected association between previous gonorrhea infection—linked to potential exposure to the antibiotics—and antimicrobial resistance, our study indicated that previous infection with gonorrhea was not associated with risk of RTA or DSC.

There are some potential limitations to this study. First, although the current study was a national surveillance survey with a large number of isolates collected from 7 provinces in China, the sample accounts for roughly only 1% of reported cases of gonorrhea during the study period in the country. Geographically, less than a quarter of the 31 provinces in the country participated in the study, and most of them were located in the economically developed areas along the coast. Moreover, the majority of male participants provided urethral specimens (99.4% of men), and the majority of female participants provided cervical or urethral specimens (96.2% of women). Our sample overrepresents men because men are usually symptomatic and easy to identify for specimen collection. Another concern is that most pharyngeal and anal gonorrhea is asymptomatic, and repeat infections or repeat visits of anonymized patients to clinics during the study period may result in the true number of individuals sampled being smaller than the number of “patients” described here, although this difference is likely to be relatively small. In addition, only a small proportion of the study participants were homosexual or bisexual, and therefore the majority of men did not report any anal or pharyngeal sexual intercourse [42]. All of these potential biases may limit the generalizability of the current study to all people infected with gonorrhea in China. Second, those isolates with MIC > 0.5 mg/l for azithromycin or MIC > 0.125 mg/l for ceftriaxone were not further categorized for susceptibility analysis to identify high-level RTA and/or resistance to ceftriaxone. Third, although molecular typing of *N*. *gonorrhoeae* was carried out in a few areas and reported previously [43], it was not performed in the present survey. Future studies should include molecular and genomic analyses to investigate gonorrhea transmission and to track the spread of antimicrobial resistance [44]. With regard to the treatment of gonorrhea in China, the national STD guidelines have not yet been updated to include dual therapy with ceftriaxone plus azithromycin for gonorrhea but recommend the use of azithromycin for treating potential infection with chlamydia among patients infected with gonorrhea. Further studies are needed to look into appropriate doses of ceftriaxone and azithromycin as well as use of other antibiotics in China.

In conclusion, the current study was a national study on the susceptibility of *N*. *gonorrhoeae* to azithromycin and ceftriaxone with implications for antibiotic choice for treatment of gonorrhea in China. An increasing trend of isolates with simultaneous RTA and DSC will likely impede the introduction of the currently WHO-recommended dual therapy in China. Therefore, evaluation of the efficacy of the dual therapy and the development of novel treatment strategies are urgently warranted in China. In addition, future resources should be dedicated to enhancing antimicrobial resistance surveillance by increasing both the number of laboratories performing susceptibility testing and the quality of susceptibility tests. The development of rapid diagnostics to promptly detect gonococcal infections and identify antimicrobial resistance is also urgently needed.

## Supporting information

S1 Checklist STROBE checklist. (DOC)

S1 Text Development of the statistical analysis plan. (DOCX)

S1 Data China-GRSP excel data for 2013–2016. (XLSX)

## Abbreviations

AOR: adjusted odds ratio
China-GRSP: China Gonococcal Resistance Surveillance Programme
CI: confidence interval
DSC: decreased susceptibility to ceftriaxone
GASP: Gonococcal Antimicrobial Susceptibility Programme
IQR: interquartile range
MIC: minimum inhibitory concentration
NCSTD: National Center for Sexually Transmitted Disease Control
RTA: resistance to azithromycin
STD: sexually transmitted disease
TM: Thayer-Martin
WHO: World Health Organization
