# Supplementary material for: Susceptibility of Neisseria gonorrhoeae to azithromycin and ceftriaxone in China: A retrospective study of national surveillance data from 2013 to 2016
Source: PLoS Med. 2018 Feb 6;15(2):e1002499. doi: 10.1371/journal.pmed.1002499 (PMC5800545; doi:10.1371/journal.pmed.1002499)
Supplement: S1 Checklist — (DOC) [file pmed.1002499.s001.doc]

**S1. STROBE Statement—checklist of items that should be included in reports of observational studies**

|  | Item No | Recommendation | Paragraph per section | | Relevant text in manuscript | |
| --- | --- | --- | --- | --- | --- | --- |
| **Title and abstract** | 1 | (*a*) Indicate the study’s design with a commonly used term in the title or the abstract | Title | | Susceptibility of Neisseria gonorrhoeae to azithromycin and ceftriaxone in China: A retrospective study of national surveillance data from 2013 to 2016 | |
|  |  | (*b*) Provide in the abstract an informative and balanced summary of what was done and what was found | Abstract | | Minimum inhibitory concentration (MIC) was determined in 3,849 isolates collected from patients with a confirmed positive N. gonorrhoeae culture at the clinic visit during the period of January 2013 through December 2016 in seven provinces in China to identify resistance to azithromycin (RTA) or decreased susceptibility to ceftriaxone (DSC) using a MIC cut-off. The prevalence of isolates with RTA was 18.6% and percentage of DSC fluctuated between 9.7% and 12.2%. The overall prevalence of the isolates with both RTA and DSC increased from 1.9% in 2013 to 3.3% in 2016 (Chi-square test for trend, P=0.03). | |
| Introduction | | |  | |  | |
| Background/rationale | 2 | Explain the scientific background and rationale for the investigation being reported | Introduction, paragraph 1 | | Gonorrhoea remains one of the most common sexually transmitted diseases (STDs) worldwide. China contributes substantially to the number of people with gonorrhoea. Effective prevention and control of gonorrhoea largely relies on detection of cases followed by therapy to cure the infection and eliminate transmission to others. However, successful treatment has been hampered by emerging resistance to each of the antibiotics recommended as first-line therapies. Cases with resistance and treatment failures to ceftriaxone have been reported and a dual therapy consisting of ceftriaxone and azithromycin has been recommended by many countries. Evidence on susceptibility to ceftriaxone and azithromycin is needed for China to update its treatment recommendations. | |
| Objectives | 3 | State specific objectives, including any specified hypotheses | Introduction, paragraph 1 | | This study was aimed to provide evidence on susceptibility of *N. gonorrhoea* isolates from China GRSP to azithromycin and ceftriaxone for updating the national treatment recommendations for gonorrhoea. | |
| Methods | | |  | |  | |
| Study design | 4 | Present key elements of study design early in the paper | Methods, paragraph 2 | | We conducted a retrospective study based on clinical isolates and data of patients with confirmed *N. gonorrhoea* in the China GRSP. | |
| Setting | 5 | Describe the setting, locations, and relevant dates, including periods of recruitment, exposure, follow-up, and data collection | Methods, paragraph 2 | | The study subjects are the patients recruited from January 2013 to December 2016 in 7 provinces (Guangdong, Guangxi, Hainan, Zhejiang, Sichuan, Tianjin, Xinjiang, Shanghai, Chongqing) in China. | |
| Participants | 6 | Give the eligibility criteria, and the sources and methods of selection of participants | Methods, paragraph 2 | | Patients who seek routine service at the sentinel STD clinics of China GRSP in the 7 provinces and who are confirmed to be infected with *N. gonorrhoea* by culture are eligible to include into the study. | |
| Variables | 7 | Clearly define all outcomes, exposures, predictors, potential confounders, and effect modifiers. Give diagnostic criteria, if applicable | Methods, paragraphs 4, 5 | | The study outcomes are resistance to azithromycin (RTA) and decreased susceptibility to ceftriaxone (DSC). The RTA is defined as having a MIC ≥ 1.0 mg/L of azithromycin while DSC is defined as having a MIC ≥ 0.125 mg/L of ceftriaxone. Prevalence rates of RTA, DSC and RTA plus DSC and their 95% confidence intervals are calculated. | |
| Data sources/ measurement | 8* | For each variable of interest, give sources of data and details of methods of assessment (measurement). Describe comparability of assessment methods if there is more than one group | Methods, paragraphs 4 | | Data of RTA and DSC come from MIC detection of isolates collected from the patients attending the sentinel STD clinics of China GRSP and data of selected demographic, behavioral and clinical characteristics are extracted from outpatient medical records. | |
| Bias | 9 | Describe any efforts to address potential sources of bias | Methods, paragraphs 2 | | We collected the clinical isolates from as many geographic areas as possible and finally included the isolates collected from 7 provinces into the study. | |
| Study size | 10 | Explain how the study size was arrived at | Methods, paragraphs 2 | | The study is based on all available clinical isolates collected by the China GRSP which is a nationwide gonococcal resistance surveillance programme. | |
| Quantitative variables | 11 | Explain how quantitative variables were handled in the analyses. If applicable, describe which groupings were chosen and why | Methods, paragraphs 5 | | Age is described as median and 25%-75% interquartile range (IQR) as the age data follow a left skewed distribution. MIC is described as geometric mean and 95% CI as MICs are multiple values determined by serial dilutions. For MICs determined as above or below a specific value, we used the midpoint value between this specific value and the value of the next higher or lower dilution for calculating the geometric means. | |
| Statistical methods | 12 | (*a*) Describe all statistical methods, including those used to control for confounding | Methods, paragraphs 5 | | Bivariate analyses are conducted by chi-square test or chi-square test for trend. Multinomial logistic regression analysis is applied to explore the associations of variables with being infected with strains of RTA, DSC or RTA/DSC. | |
|  |  | (*b*) Describe any methods used to examine subgroups and interactions | Methods, paragraphs 5 | | Multinomial logistic regression analysis is applied. | |
|  |  | (*c*) Explain how missing data were addressed |  | | NA | |
|  |  | (*d*) *Cross-sectional study*—If applicable, describe analytical methods taking account of sampling strategy |  | | NA | |
|  |  | (*e*) Describe any sensitivity analyses |  | | NA | |
| Results | | |  |  | |  |
| Participants | 13* | (a) Report numbers of individuals at each stage of study—eg numbers potentially eligible, examined for eligibility, confirmed eligible, included in the study, completing follow-up, and analysed |  | | NA | |
|  |  | (b) Give reasons for non-participation at each stage |  | | NA | |
|  |  | (c) Consider use of a flow diagram |  | | NA | |
| Descriptive data | 14* | (a) Give characteristics of study participants (eg demographic, clinical, social) and information on exposures and potential confounders | Results, paragraph 1 | | A total of 3,849 N. gonorrhoeae isolates were collected from 3,849 patients (males, 91.1%; females, 8.9) and no homosexuals were found to have positive *N. gonorrhoea* isolate at more than one anatomical site. Of the 3,503 males who provided information on sexual orientation (99.9% of the male total), 3,451 (98.5%) were heterosexual and 52 (1.5%) were homosexual or bisexual. The median age was 31 years (IQR, 26-40 years) for males and 31 years (IQR, 25-43 years) for females. The majority (92.8%) of the participants were of Han ethnicity and 50.2% were from the provinces along the coast. | |
|  |  | (b) Indicate number of participants with missing data for each variable of interest | Results, paragraph 2 | | Out of 3,849 *N. gonorrhoeae* isolates, 3,827 (99.4%) is with MIC result of azithromycin. | |
| Outcome data | 15* | *Cross-sectional study—*Report numbers of outcome events or summary measures | Results, paragraphs 2, 3 | | 3827 isolates provide with MIC results of azithromycin and 3849 with the results of ceftriaxone for determining RTA and DSC, respectively. The RTA and DSC rates are 18.6% and 10.8%, respectively. | |
| Main results | 16 | (*a*) Give unadjusted estimates and, if applicable, confounder-adjusted estimates and their precision (eg, 95% confidence interval). Make clear which confounders were adjusted for and why they were included | Results, paragraphs 2, 3; S2 | | The unadjusted prevalence of RTA is 18.6% (710/3827; 95% CI, 17.4%-19.8%) while the prevalence of DSC is 10.8% (417/3849; 95% CI, 9.9%-11.9%). Based on the bivariate analyses, we included five independent variables into the logistic regression analyses: age (continuous variable); sex, history of gonococcal infections, sexual orientation and residence along the coast (dichotomous variables). We included the interaction between sex and sexual orientation in the logistic regression analyses. | |
|  |  | (*b*) Report category boundaries when continuous variables were categorized | Results, paragraph 1 | | For MICs determined as above or below a specific value, we use the midpoint value between this specific value and the value of the next higher or lower dilution for calculating the geometric means. | |
|  |  | (*c*) If relevant, consider translating estimates of relative risk into absolute risk for a meaningful time period |  | | NA | |
| Other analyses | 17 | Report other analyses done—eg analyses of subgroups and interactions, and sensitivity analyses |  | | NA | |
| Discussion |  |  |  | |  | |
| Key results | 18 | Summarise key results with reference to study objectives | Discussion, paragraph 1 | | Our findings indicate that the rate of dual resistance to azithromycin and decreased susceptibility to ceftriaxone increased between 2013 and 2016. Resistance of *N. gonorrhoeae* to azithromycin and decreased susceptibility to ceftriaxone in China were higher than many countries. | |
| Limitations | 19 | Discuss limitations of the study, taking into account sources of potential bias or imprecision. Discuss both direction and magnitude of any potential bias | Discussion, paragraph 4 | | Several potential limitations to this study are highlighted. First, the current study had a relatively small and limited geographic coverage and population in the country. Second, MICs were not further categorized to identify high-level resistance to azithromycin and/or resistance to ceftriaxone. Third, molecular typing was not performed in the present survey. | |
| Interpretation | 20 | Give a cautious overall interpretation of results considering objectives, limitations, multiplicity of analyses, results from similar studies, and other relevant evidence | Discussion, paragraph 1 | | Our findings on RTA and DSC were higher than many countries including those in the Europe and the Americas. However, Japan has also had a high prevalence of resistance. It is unclear whether circulating gonococcal strains with altered susceptibility to the key antibiotic agents in China acquired these mutations through domestic selective pressure or whether the strains have been imported. | |
| Generalisability | 21 | Discuss the generalisability (external validity) of the study results | Discussion, paragraph 4 | | All of these potential biases may limit the representativeness of the current study to all people infected with gonorrhoea in China. | |
| Other information |  |  |  | |  | |
| Funding | 22 | Give the source of funding and the role of the funders for the present study and, if applicable, for the original study on which the present article is based | Methods, paragraph 1 | | The China-GRSP has been funded by the National Health and Family Planning Commission of China. The data analysis and manuscript preparation work were supported by grant from the Chinese Academy Medical Sciences Initiative for Innovative Medicine (2016-I2M-3-021). | |

*Give information separately for cases and controls in case-control studies and, if applicable, for exposed and unexposed groups in cohort and cross-sectional studies.

**Note:** An Explanation and Elaboration article discusses each checklist item and gives methodological background and published examples of transparent reporting. The STROBE checklist is best used in conjunction with this article (freely available on the Web sites of PLoS Medicine at http://www.plosmedicine.org/, Annals of Internal Medicine at http://www.annals.org/, and Epidemiology at http://www.epidem.com/). Information on the STROBE Initiative is available at www.strobe-statement.org.
