# Supplementary material for: Susceptibility of Neisseria gonorrhoeae to azithromycin and ceftriaxone in China: A retrospective study of national surveillance data from 2013 to 2016
Source: PLoS Med. 2018 Feb 6;15(2):e1002499. doi: 10.1371/journal.pmed.1002499 (PMC5800545; doi:10.1371/journal.pmed.1002499)
Supplement: S1 Text — (DOCX) [file pmed.1002499.s002.docx]

**S1 Text. Development of the Statistical Analysis Plan**

1. Study context

Data for the current study come from the China Gonococcal Resistance Surveillance Programme (China-GRSP) which was established as a study project in 1987 and then expanded to a nationwide programme in 2007. As part of the World Health Organization (WHO), the China-GRSP follows the WHO Methodologies in Gonococcal Antimicrobial Surveillance Programmes (GASPs) [1] to conduct the minimum inhibitory concentration (MIC) detection with clinical isolates using standard antibiotics provided by the WHO. The China-GRSP regularly participate in WHO proficiency testing and quality control for MIC detection.

The current study is a retrospective analyses of data from the China-GESP that was conceptualized after the detection of clinical isolates for MICs to six antimicrobials (penicillin, tetracycline, ciprofloxacin, azithromycin, cefixime and ceftriaxone) and entry of characteristic data and MIC data were completed. We were interested in describing the conditions in susceptibilities of *N. gonorrhoeae* to the two antimicrobials (azithromycin and ceftriaxone) which are currently recommended by WHO to include into the dual therapy for treatment of gonococcal infections. We were also interested in testing the hypothesis that occurrence of resistance to azithromycin and decreased susceptibility to ceftriaxone changes over year and there are independent factors associated with the resistance to azithromycin or/and decreased susceptibility to ceftriaxone. As MIC detection of the clinical isolates for azithromycin was initiated at national level in 2013 although a pilot study in two cities was conducted by us between 2008 and 2009 [2], the current study is based on analyses of the data between 2013 and 2016.

1. Analysis plan prior to commencing the current manuscript

The analyses presented in the current manuscript evolved from the following research question and associated statistical analysis:

1. What are the determinants used to describe the susceptibility of *N. gonorrhoeae* to antimicrobials?
   1. We will use the WHO criteria to categorize susceptibility to azithromycin as “not resistance (MIC ≤ 0.5 mg/L)” and “resistance (MIC≥ 1.0 mg/L)” and susceptibility to ceftriaxone as “not decreased susceptibility (MIC ≤ 0.06 mg/L)” and “decreased susceptibility (MIC ≥ 0.125 mg/L)”.
   2. We will also use constituent ratios of different MIC levels during 2013 and 2016 to show the evolutions of MIC levels over the time.
2. How will we measure the spread of the strains with resistance and/or decreased susceptibility to specific antimicrobial in a population?
   1. We will use one isolate per patient for assessment of the antibiotic susceptibility.
   2. We will calculate the prevalence rates of “resistance to azithromycin” and/or “decreased susceptibility to ceftriaxone” and their 95% confidence intervals (CIs) to estimate frequencies of the events related to the resistance among the study population.
   3. We will also calculate the proportions of different MIC levels in the study population in each of years to indicate the constituent changes of MIC levels over time.
3. Which factors are independently associated with “resistance to azithromycin” or/and “decreased susceptibility to ceftriaxone”?
   1. We will use “resistance to azithromycin” and/or “decreased susceptibility to ceftriaxone” as dependent variables for the analyses.
   2. We will use bivariate analyses to test associations between each of characteristic (independent) variables (demographic, behavioral and clinical characteristics) and each of the dependent variables. Independent variables with significance level of p<0.10 will be included in multivariate logistic regression model to explore the association of indicators with acquisition of the resistance and/or decreased susceptibility.
   3. We will use multivariate logistic regression models to identify the independent variables at a significant level of p≤0.05 and estimate adjusted odds ratio (AOR) and its 95% CIs by adjusting for potential confounding factors.
4. Changes to the analysis plan after getting comments from peer-reviewers

Based on comments and suggestions from one of the peer-reviewers to recommend application of multinomial logistic regression, the following revisions to the analysis plan were made to improve suitability and rationality of the data analyses:

1. We kept dichotomizing the dependent variable into "resistance or decreased susceptibility" and "not resistance or decreased susceptibility" but we classified the conditions (dependent variables) into four levels: (1) resistance to azithromycin and decreased susceptibility to ceftriaxone, (2) resistance only to azithromycin, (3) decreased susceptibility only to ceftriaxone and (4) neither resistance to azithromycin nor decreased susceptibility to ceftriaxone.
2. We used multinomial logistic regression to identify the independent variables significantly associated with each of the above dependent variables.
3. We combined the subjects with homosexual or bisexual orientation for the logistic regression analyses due to the small number of subjects reporting homosexual or bisexual behaviors (<2%).
4. Based on the bivariate analyses, we included five independent variables into the logistic regression analyses: age (continuous variable); sex, history of gonococcal infections, sexual orientation and residence along the coast (dichotomous variables).
5. We included the interaction between sex and sexual orientation in the logistic regression analyses.
6. Wi T, Lahra MM, Ndowa F, et al. Antimicrobial resistance in *Neisseria gonorrhoeae*: Global surveillance and a call for international collaborative action. PLoS Med. 2017; 14:e1002344.
7. Yuan LF, Yin YP, Dai XQ, et al..Resistance to azithromycin of *Neisseria gonorrhoeae* isolates from 2 cities in China. Sex Transm Dis. 2011; 38:764-8.
